# Multisystem Symptoms in Myotonic Dystrophy Type 1: A Management and Therapeutic Perspective

**DOI:** 10.3390/ijms26115350

**Published:** 2025-06-02

**Authors:** Dhvani H. Kuntawala, Rui Vitorino, Ana C. Cruz, Filipa Martins, Sandra Rebelo

**Affiliations:** Medical Sciences Department, Institute of Biomedicine—iBiMED, University of Aveiro, 3810-183 Aveiro, Portugal; dhvani.kuntawala@ua.pt (D.H.K.); rvitorino@ua.pt (R.V.); anacatarinacruz@ua.pt (A.C.C.); samartins@ua.pt (F.M.)

**Keywords:** rare disease, myotonic dystrophy type 1, multisystem disease, disease-modifying therapy, clinical trials, muscular dystrophy

## Abstract

Myotonic dystrophy type 1 (DM1) is a complex, multisystemic neuromuscular disorder with several pathological phenotypes, disease severities and ages of onset. DM1 presents significant challenges in clinical management due to its multisystemic nature, affecting multiple organs and systems beyond skeletal muscle. Tackling this condition requires a comprehensive approach that goes beyond symptom management, particularly considering the complexity of its manifestations and in the delayed diagnosis. In this review we will discuss the multisystem symptoms of DM1 and how this understanding is guiding the development of potential therapies for the improvement of patient outcomes and quality of life. This review aims to explore the available treatments and potential novel disease-modifying therapies targeting DM1 molecular mechanisms to address the broad multisystem symptoms of DM1. Effective strategies to manage symptoms remain crucial, such as physical therapy, medications for myotonia and diligent cardiac care. Metabolic management and hormonal therapies play crucial roles in addressing endocrine and metabolic abnormalities. Nevertheless, promising targeted therapies that include antisense oligonucleotides (ASOs) for RNA degradation, small molecules to disrupt protein-RNA interactions and gene editing offer a prospective approach to the underlying mechanisms of DM1 and improve patient outcomes across the different organ systems.

## 1. Introduction

**Figure 1:**
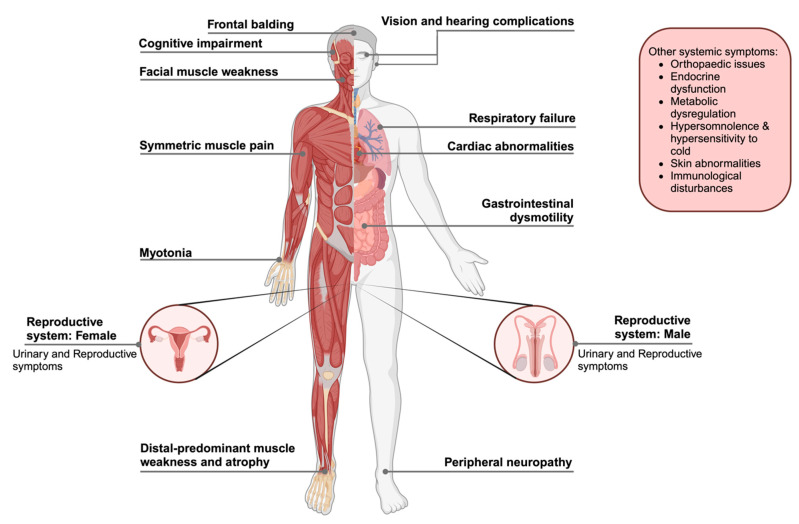
Broad spectrum symptoms of myotonic dystrophy type 1 (DM1). The figure shows the multisystem effects of DM1, emphasizing the involvement of the muscular, cardiovascular, respiratory, gastrointestinal, neurological and reproductive systems. It also highlights neurological and endocrine dysfunctions linked to the disease, demonstrating the widespread and complexity of DM1 in the body. Figure created with BioRender (BioRender.com).

Myotonic dystrophy type 1 (DM1), also known as Steinert’s disease (OMIM#160900), is the most common form of muscular dystrophy among adults, although it affects both adults and children [1]. DM1 is an autosomal dominant disease caused by unstable expanded cytosine–thymine–guanine (CTG) repeats within the 3′ untranslated region (3′ UTR) of the *DMPK* (*Dystrophia Myotonica Protein Kinase*; OMIM* 605377) gene at chromosome 19q13.3 [2,3,4,5]. Although DM1 present high inter- and intra-individual variability [6], the cardinal symptoms are myotonia often affecting the hands, tongue and jaw due to slow muscle relaxation [7], distal progressive muscle wasting and weakness, facial and bulbar involvement, high palate and dysphonia [8]. DM1 prevalence estimates differ across different geographic and ethnic groups, and delayed and missed diagnoses make it challenging to determine the real prevalence of DM1 [9,10]. Globally, DM1 has a reported prevalence of 9.27 cases per 100,000 individuals [11] and is estimated to affect between 1 in 2100 and 1: in 8000 people [12], making it one of the most common rare diseases. Widespread chronic pain affecting the hands and feet suggests an involvement of small and large fiber neuropathy [13,14]. The disease displays a multisystemic complexity, affecting several body systems, besides the skeletal muscle, such as the heart [15,16,17], the eye [18], the respiratory system [19,20,21], the endocrine system [22,23] and the central nervous system (CNS) [24,25,26] (Figure 1). Beyond the primary symptoms, DM1 patients have an increased risk for both benign tumors and malignancies [27,28,29,30] and higher complications of adverse reactions to anesthesia and analgesic drugs relative to the general population [31]. To this date, there are no disease-modifying therapies available for DM1.

## 2. Myotonic Dystrophy Type 1 Classification

The hallmark of DM1 is the abnormal expansion of the CTG trinucleotide repeats beyond a normal range. In stable, unaffected individuals, the length of the CTG expansion ranges from five to thirty-four repeats, which are stably inherited and have a low mutation rate. DM1 patients have CTG expansions exceeding 50 repeats and extending from hundreds to thousands of repeats becoming highly unstable and often expanding further in both germline and somatic cells. DM1 alleles also include a ‘premutation range’ with CTG repeat lengths ranging from 38 to 49 [32,33]. Individuals with permutations show none or few mild symptoms, but are more likely to pass a pathologically expanded mutant allele to the next generations [34]. DM1 displays the phenomenon of anticipation, characterized by larger repeat expansions from one generation to the next, resulting in an earlier onset and more pronounced clinical features [9].

**Figure 2:**
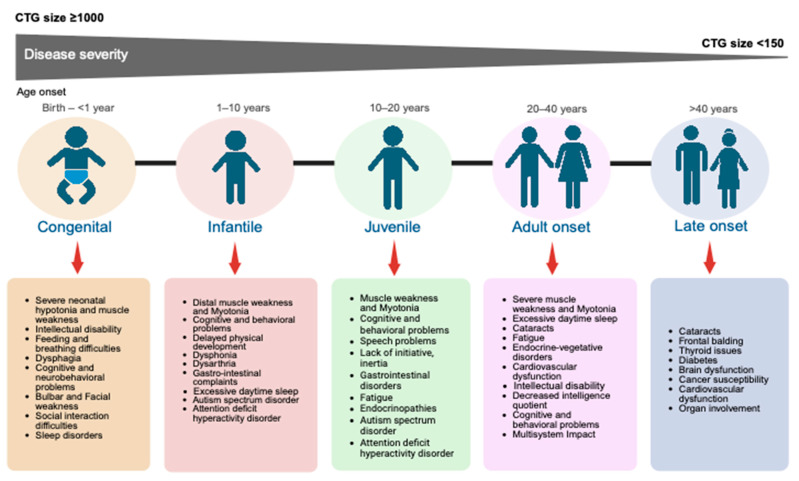
Myotonic dystrophy type 1 (DM1) phenotypes and clinical manifestations. The grey arrow illustrates the overall disease severity trend across the DM1 subtypes: as CTG repeat sizes increase, both disease severity and symptom complexity tend to worsen, often appearing earlier in life. Each phenotype is associated with a characteristic set of symptoms, ranging from mild manifestations in lower repeat ranges to severe and multisystemic involvement as the repeat length increases [38,51,52,53,54,55,56,57,58]. Figure created with BioRender (BioRender.com). Abbreviations: DM1, myotonic dystrophy type 1; CTG, cytosine-thymine-guanine. These clinical features have been oversimplified for comparison purposes.

From the clinical perspective, DM1 is typically classified according to the age of onset and the severity of disease symptoms. DM1 is classified into three phenotypes (mild, classic and severe) based on severity and clinically categorized as congenital-onset, infantile-onset, juvenile-onset, adult-onset and late-onset [35]. Each phenotype is associated with CTG repeat expansions larger than 100 and up to several thousands, with major overlap in CTG repeat sizes across different phenotypes [24,36,37] (Figure 2).

Congenital-onset DM1 is the most severe form and is characterized by a CTG repeat length greater than 1000 repeats [35,37]. The first clinical symptoms appear prenatally or at birth, with an estimated incidence ranging from 2 to 28 per 100,000 live births [38]. In the ‘severe’ form, also referred to as congenital DM1, symptoms can include hypotonia, generalized muscle weakness and myopathic facies, which may result in feeding difficulties and require respiratory support [39]. In contrast to juvenile- and adult-onset, myotonia is typically absent in congenital DM1 in early childhood [40].

The first clinical symptoms in infantile-onset DM1 appear around the ages one to ten. Although symptoms can develop after the first year of life, they are often more noticeable in early childhood, with initial signs being cognitive or behavioral rather than physical. This can include intellectual disabilities and attention deficits, which could complicate early diagnosis [41,42]. In juvenile-onset, the first clinical symptoms are detected between ages 10 and 20; this form usually presents with a broader range of physical symptoms particularly muscle weakness [43]. Both infantile-onset and juvenile-onset conditions develop symptoms like those noticed in adults and normally present more than 400 CTG-repeats [44,45]. Adult-onset or ‘Classic DM1 form’ is the most common type of DM1, typically developing between the ages of 20 and 40 [46]. This type is associated with CTG repeat expansions ranging from 50 to less than 1000 repeats and has a global prevalence of 9.27 per 100,000 [11]. This form has been associated with premature aging, with signs of early cognitive decline and, in some cases, dementia [47,48]. In support of this, longitudinal studies show progressive cognitive decline and changes in brain white matter. These studies also reveal that the longer the disease lasts, the more severe the neurocognitive dysfunction becomes, signifying worsening brain function over time [49]. Finally, the late-onset, also known as ‘Mild DM1 form’, typically starts after the age of 40 and is characterized by mild distal muscle weakness and cataracts [50]. These patients typically have a CTG repeat size of 50 to 100 and have a normal or only minimally shortened lifespan [46].

## 3. Myotonic Dystrophy Type 1 Molecular Pathophysiology

**Figure 3:**
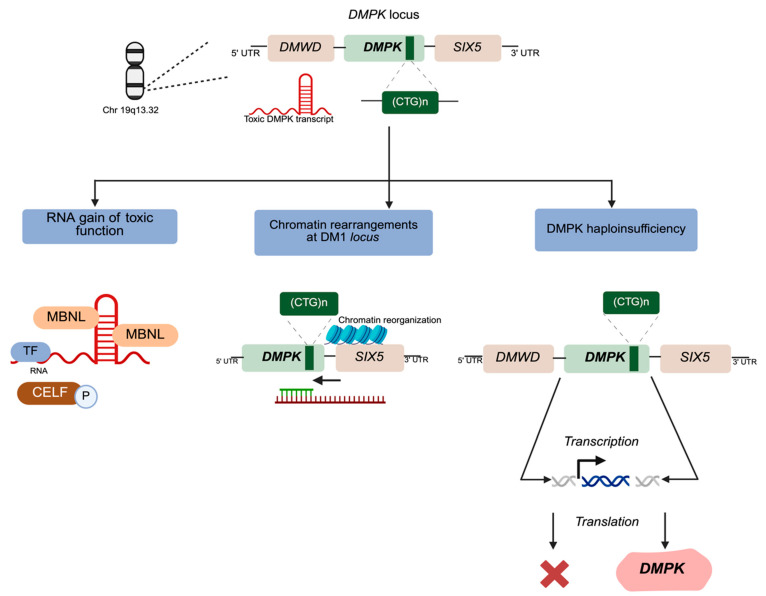
Proposed models of DM1 pathological mechanisms. The CTG expansion affects chromatin rearrangement, leading to epigenetic repression of neighboring genes. Once transcribed into RNA, these repeat expansions exert a toxic RNA gain-of-function effect within the cells. The CTG repeat expansion in the *DMPK* gene interferes with normal gene expression by altering transcription or by causing the retention of CUG expanded transcripts, which may lead to DMPK haploinsufficiency, contributing to disease pathology. Figure created with BioRender (BioRender.com) Abbreviations: DMPK, myotonic dystrophy protein kinase; DMWD, DM1 locus WD repeat containing; SIX, homeobox gene; (CTG)n, expansion of (cytosine-thymine-guanine) n; mRNA, messenger ribonucleic acid; CELF, CUGBP Elav-like family; MBNL1, muscleblind-like splicing regulator 1; miRNA, micro-RNA; circRNA, circular RNA; RAN, ras-related nuclear protein.

To date, the pathological mechanisms underlying DM1 pathogenesis remain partially understood. Some models have been proposed to explain the pathological mechanisms for DM1, namely the chromatin rearrangement, RNA toxicity and DMPK haploinsufficiency models (Figure 3). However, currently, no single model accounts for the full spectrum of clinical symptoms observed in DM1 patients.

Concerning chromatin rearrangement at the DM1 locus (Figure 3), the transcription of the *DMPK* gene yields into sense and anti-sense transcripts, playing important roles in the regulation of gene expression and the pathogenic mechanisms associated with the disease. Processing of anti-sense transcript might be involved in chromatin ultrastructure regulation and may extend into an insulator element found between the *DMPK* and *SIX5* genes. Double-stranded RNA structures formed either by CUG transcripts folding into hairpin structures or complementary binding of sense and anti-sense DMPK transcripts, may activate RNA interference (RNAi) pathways [59,60,61]. The repressive chromatin environment may lead to reduced transcription of DMPK and adjacent genes, contributing to disease pathology.

Considering the RNA gain of toxic function (Figure 3), the mutant DMPK RNA containing expanded CUG repeats (CUGexp) accumulates in the nucleus, building ribonuclear foci with RNA hairpin structures that trap RNA-binding proteins (RBPs) important for mRNA transport, alternative splicing and decay. Muscleblind-like protein 1 (MBNL1) and CUG-BP-ELAV-like family member 1 (CUGBP1 or CELF1) are key RBPs affected. The sequestration of MBNL proteins by DMPK CUGexp mRNA within the nucleus leads to a reduction in their functional activity and availability in the cell [62]. A significant result of MBNL1 sequestration is the interference with its typical function in the alternative splicing of transcripts related to mitochondrial structure and dynamics, like optic atrophy 1 (OPA1) and Mitofusin-2 (MFN2), which are essential for mitochondrial fusion [63,64]. Alterations in the splicing of these genes lead to an increase in mitochondrial fission, resulting in abnormal mitochondrial structures marked by fragmentation, swelling, disorganized cristae and clustering around the nucleus [65,66]. These structural abnormalities are associated with aberrant mitochondrial performance, which includes decreased ATP production, heightened oxidative stress, and ineffective mitophagy, factors that contribute to the cellular dysfunction seen in DM1 [67,68].

In contrast, CUGBP1 is not sequestered in a similar manner but is instead hypothesized to undergo hyperphosphorylation mediated by several protein kinases, such as protein kinase C (*PKC*), cyclin D3 (*CCND3*), cyclin-dependent kinase 4 (*CDK4*), AKT serine/threonine kinase 1 (*AKT1*), glycogen synthase kinase 3 beta (*GSK3B*) and double-stranded RNA-dependent protein kinase (*PKR*) [69,70]. These kinases modify CUGBP1 by affecting its stability, localization and activity. Hyperphosphorylation of CUGBP1 enhances its activity, leading to dysregulation in alternative splicing, mRNA translation and transcript decay [71]. This aberrant regulation disrupts normal cellular functions and contributes to the molecular pathology of DM1, even though CUGBP1 itself is not directly sequestered by toxic RNA repeats [72].

The symptoms observed in DM1 patients are believed to be caused by MBNL and CUGBP1 dysfunctions. In the nucleus, the misregulation of splicing factors MBNL and CUGBP1 interrupts the normal developmental splicing process, preventing the suitable switch from embryonic splice isoforms to adult-specific isoforms in adult tissues [73]. Misspliced effector genes, such as muscle-specific chloride channel (*ClC1*), NMDA receptor (*NR1*), insulin receptor (*IR*), cardiac troponin T (*cTNT*), MTMR1 and Tau (*MAPT*) have been identified as key targets of *MBNL* and *CELF1*. The dysregulation of these genes due to mis-splicing contributes to the multisystemic symptoms observed in DM1 [74,75]. Mutant *DMPK* RNA interferes with the function of specific transcription factors (TFs), depleting them from active chromatin through an RNA leaching mechanism, and thereby demonstrating a decreased level of transcription from the CLCN1 promoter [76,77,78].

DMPK haploinsufficiency (Figure 3) caused by the CTG expansion results in an approximately 50% reduction in DMPK expression, either due to altered transcription or the retention of CUG-expanded transcripts, leading to disease pathology [79,80]. DMPK plays a crucial role in the phosphorylation of proteins like sarcolipin (SLN), phospholamban (PLB), phospholemman (PLM) and Lipin, which are necessary for muscle contraction and relaxation [35]. It is reasonable to conclude that protein phosphorylation plays a key regulatory role in DM1 [81]. In fact, phosphorylated PLB and SLN interact with Sarco/Endoplasmic reticulum Ca^2+^-ATPase (SERCA)*,* leading to elevated calcium levels in the cytoplasm. This results in prolonged muscle contraction, which causes myotonia in DM1 [82,83].

Despite all this information, none of these theories can account for all the multisystemic symptoms. However, more recent research has shed light on additional events that could contribute to the disease complexity. Among these, repeat-associated non-ATG (RAN) translation plays a central role in disease pathology by generating toxic non-physiological peptides or proteins through bidirectional transcription and several reading frames [84,85]. This aberrant RNA accumulation contributes to cellular toxicity and triggers apoptotic pathways by forming nuclear RNA foci and accumulating in the cytoplasm [86], resulting in toxicity that contributes to the multisystemic manifestations seen in DM1. The presence of RAN-translated proteins has been seen in multiple DM1 cell types, although their detection remains challenging due to low expression levels compared to controls [87,88]. The understanding of RAN translation’s role in DM1 continues to progress, emphasizing its potential impact on disease progression.

Beyond RAN translation, microRNA (miRNA) changes have been implicated in DM1 pathogenesis [80]. Aberrant expression of miRNAs in DM1 disrupts normal gene expression and regulatory networks, which may intensify disease progression [89]. Altered signaling pathways, such as those involving transforming growth factor-beta (TGF-β) [90], extracellular signal-regulated kinase (ERK) [91,92] and AMP-activated protein kinase (AMPK) [93], underscore the complexity of the molecular landscape in DM1. These disruptions in signaling pathways are often directly or indirectly influenced by the toxic effects of expanded CUG repeats [94].

Emerging research also points the dysregulation of nuclear envelope (NE) integrity and function as a significant contributor to DM1 pathogenesis [95]. Intriguingly, primary DM1 myoblast and myotube cultures have shown changes in the expression of NE transmembrane proteins (NETs) and modifications to the structure of the NE [96,97]. A study using DM1 patient-derived fibroblasts showed abnormalities in NE structures, including altered levels and mislocalization of NE proteins (Lamin A/C, LAP1, SUN1, nesprin-1 and nesprin-2). These changes were accompanied by a high prevalence of micronuclei, increased number of nuclear inclusions and nuclear deformations (blebs, lobes, invaginations). These results emphasize NE dysfunction as a pathological feature in DM1 [98].

Additionally, genome organization, regulation, repair, signaling and cellular mechanics are all significantly impacted by the NE [99,100]. For instance, variations in the nuclear pore complex function caused by defective NETs can hinder mRNA export, contributing to the nuclear retention of toxic RNA species [101,102,103].

Furthermore, mitochondrial dysfunction [67,104] and oxidative stress [105,106] are gradually being recognized as contributing factors to DM1 pathophysiology.

## 4. Multisystem Symptoms Management and Overview of the Current Clinical Treatments

### 4.1. Musculoskeletal System

Patients with DM1 present muscle weakness with a characteristic pattern, affecting both distal and facial muscles, with proximal muscles becoming involved as the disease progresses. While myotonia is the hallmark of the disease, it is generally mild to moderate and may not always require treatment [107]. However, some patients experience severe and disabling myotonia, which significantly impacts their quality of life, leading to fatigue, pain, droopy eyelids, handshaking and breathing and swallowing difficulties [108]. In such cases, anti-myotonic drugs may be used, such as mexiletine, which significantly reduces hand grip myotonia in DM1 patients with no serious events [109,110,111], although no substantial outcome was observed in the 6 min walk test [110]. The results from two ongoing clinical trials revealed that administration of mexiletine in DM1 patients, three times daily at dosages of 150–200 mg, has demonstrated to be both effective and well-tolerated, leading to a reduction in handgrip relaxation time [111]. The Myotonic Dystrophy Foundation (MDF) advocates mexiletine as a consensus-based care recommendation for adults with DM1 and a safe first-choice anti-myotonic medication for reducing muscle stiffness [112]. Given the risk of pro-arrhythmogenic effects, an ECG should be conducted before starting therapy, followed by regular ECGs and clinical supervision by cardiologists [113].

Another important aspect of this disease is the progressive muscle wasting or atrophy that presents a distal pattern, affecting the muscles of arms, hands, ankles, jaw, tongue and neck flexors. In advanced stages of DM1, mobility assistive devices such as powered wheelchairs and motorized scooters are usually utilized to help patients with muscle deterioration in the daily living activities [114,115]. Further, a fundamental aspect of managing muscle weakness in DM1 involves a regular assessment of functional independence, respiratory muscle weakness, mobility disturbances, dysphagia and speech difficulties [116].

Although multiple drugs are being tested, few have shown reliable improvements in muscle function and strength in patients with DM1 [117,118,119,120]. Activating AMPK through combinatorial therapies for rescuing skeletal muscle defects and maximizing therapeutic benefits presents a favorable strategy for improving muscle function in DM1 [121,122]. Moreover, incorporating regular aerobic exercises at low to moderate intensity to improve mobility [123], along with the support from speech, physical and occupational therapists or orthopedic treatments as required, is essential for enhancing patients’ quality of life, despite the lack of evidence-based conclusions [112,116,124,125]. Accurately measuring muscle strength is crucial for understanding the progression of DM1, which impacts both cardiac and skeletal muscles. The most used methods for assessing cardiac muscle strength include echocardiography and the measurement of ejection fraction [126]. In contrast, skeletal muscle strength is typically evaluated through quantitative muscle testing, manual muscle testing, maximum isometric torque and the Medical Research Council (MRC) scale. These tools guide therapeutic intervention and provide insights into the extent of muscle involvement [127].

### 4.2. Respiratory System

Respiratory failure is the primary cause of mortality in patients with DM1 [128], often resulting from a combination of central respiratory drive issues, skeletal muscle weakness and upper airway muscle dysfunctions, leading to obstructive sleep apnea and aspiration [129,130,131]. However, many healthcare providers may overlook DM1 as a possible underlying cause, particularly in individuals without a clear history of respiratory issues [129,132]. Chronic respiratory difficulties affect about 30% of the DM1 population making it a life-threatening disorder [36,133]. Obstructive sleep apnea appears in 52–86% of patients and central apnea appears in 44%, along with sleep-associated hypoventilation and hypoxemia [133,134,135], all of which subsidize to an increased rate of cardiac irregularities [136,137]. A significant number of patients with DM1 present a high prevalence of sleep disorders, with most cases being obstructive in nature [138]. In fact, by using polysomnographic test and multiple sleep latency test (MSLT), several notable polysomnographic abnormalities were recognized in DM1 patients compared to controls [139]. Chronic respiratory insufficiency, with or without sleep apnea, can be effectively managed using non-invasive home mechanical ventilation (HMV), which has been shown to relieve symptoms, providing an opportunity of survival benefit to patients [140]. On the other hand, a large observational study hypothesized that adherence to HMV treatment in DM1 patients is not consistently associated to their respiratory characteristics, making it unreliable for those at risk because of low adherence [141].

Upon diagnosis, DM1 patients should receive baseline respiratory function tests and undergo annual assessments of vital capacity in both sitting and supine positions [142]. For patients with dysphagia, impaired cough or compromised lung function, it is crucial to monitor for recurrent respiratory infections and consider the use of cough assistance devices [44,112,142]. Screening for sleep-disordered breathing is necessary, with non-invasive ventilation (NIV) being considered if extreme daytime sleepiness (EDS) is present and ventilation criteria are met [143]. For patients who do not respond to NIV, medications such as modafinil, methylphenidate and a theobromine/caffeine combination have demonstrated effectiveness and good tolerance in treating EDS [143,144,145]. Additional research into the natural progression of lung dysfunction in DM1 patients will help assess risks, provide insights into the disease’s pathogenesis and develop suitable outcome measures and treatments for upcoming therapeutic trials.

### 4.3. Cardiovascular System

Cardiac involvement is observed in approximately 80% of DM1 cases [146,147], often preceding muscular impairment. The cardiac alterations in DM1 include conduction disturbances, arrhythmias and subclinical diastolic and systolic dysfunction in the earliest stages of the disease [148,149,150,151]. Severe ventricular systolic dysfunction typically occurs later with the disease progression. Although dilated and end-stage cardiomyopathy are rare in DM1, myocardial infarction accounts for around 5% of cardiovascular deaths, and unexpected cardiac death occurs in 30% of patients [148,151].

Clinical presentations may include chest pain, pre-syncope, dyspnea and palpitations [152].

Despite significant advances in understanding the pathophysiology of skeletal muscle in DM1, the mechanisms underlying cardiac arrhythmogenic aspects remain unclear [153]. Initial screening for symptomatic individuals consists of an electrocardiograph, echocardiogram and Holter ECG monitorization [154]. In cases where surface ECGs reveal uncertain abnormalities, invasive electrophysiological studies can be valuable [155]. Detecting myocardial structural and functional abnormalities is possible through non-invasive use of contrast-enhanced cardiac MRI [147,156].

Implantable cardioverter defibrillators (ICDs) have proven to offer a survival benefit in selected patient groups, particularly those with high risk of sudden cardiac death due to inherited arrhythmias, heart failure or previous cardiac arrest [157]. However, recent advancements in heart failure therapy and clinical trials have sparked a renewed interest and raised emerging questions about the role of primary prevention ICDs, particularly in individuals with non-ischemic heart failure, in reducing mortality and improving patient outcomes [158].

Additionally, pacemakers may be used in DM1 patients to treat symptomatic bradyarrhythmia or prophylactically in those at an elevated risk of complete heart block [159]. The use of class 1 antiarrhythmic drugs in DM1 patients should be approached with caution due to their paradoxical proarrhythmogenic effects [155]. Mexiletine is also encouraged by cardiologists occasionally to relieve atrial fibrillation [160]. A thorough evaluation must be carried out before initiating the use of an anti-arrhythmics in patients with DM1 to rule out any underlying abnormalities. Therefore, cardiologists with experience in treating DM1 patients should oversee monitoring during drug initiation and the mexiletine-related monitoring [111,161]. An algorithm has been developed by cardiologists and neurologists to guide decision-making regarding mexiletine treatment and conducting cardiac monitoring [161]. The guidelines for permanent pacemaker implantation in DM1 patients with cardiac conduction disturbances are like those for the general population [162]. Although, some DM1 patients who do not meet the formal criteria have depicted better outcomes with an invasive strategy, highlighting the necessity for risk stratification and personalized care and the importance of developing DM1-specifc guidelines [163]. When treating DM1 patients, cardiologists should recognize preclinical echocardiographic indicators of heart impairment to identify those who require intensive therapeutic management in a timely manner [149]. Additionally, regardless of cardiological symptoms, yearly ECGs are encouraged in DM1 patients [112].

### 4.4. Gastrointestinal System

Smooth musculature is also impacted by myotonia and weakness, leading to the prevalence of gastrointestinal (GI) symptoms that can affect the DM1 patient’s quality of life. These patients frequently present a variety of GI symptoms, affecting all regions of the GI tract, including liver and gallbladder, esophagus, small and large intestines, stomach and anal sphincter [164,165]. Dysphagia (difficulty in swallowing), is the most common GI complaint [166], resulting from myotonia and weakness of the oropharyngeal muscles and/or impaired esophageal motility [167]. Other frequent symptoms include acid reflux, intestinal pseudo-obstruction, variations between diarrhea and constipation that can mimic irritable bowel syndrome, as well as gallbladder problems with a comparatively high incidence of cholecystectomy and liver complications [165,168,169]. These GI manifestations result from a combination of several factors, including connective tissue alterations, smooth muscle dysfunction [48], autonomic neuropathy and neuromuscular impairment [14]. Some studies suggested a possible correlation between the CTG repeat length and the GI signs such as constipation, dysphagia and abdominal discomfort [166,170]. Emerging research also indicates that the gut bacterial community, in combination with biochemical factors, may influence the GI tract, even though no significant changes in the gut microbiome composition have been observed between the different DM1 phenotypes [164]. This happens because of the microbiome’s functional activity, metabolic pathways and interactions with immune responses or metabolic byproducts that could impact GI health without altering its overall configuration [171]. These complex interactions highlight that even in the absence of significant compositional changes, the gut bacterial community can still contribute to GI manifestations in DM1 through functional or metabolic mechanisms [172]. Beyond microbiome composition, there is need to fully understand its role in DM1-associated GI dysfunction.

Clinicians should closely observe for symptoms such as frequent coughing, weight loss, dysphagia and dysphonia [112,173]. If necessary, dietary adjustments and swallowing techniques should be effectively taught to the patients [174,175], along with rehabilitation approaches to avoid aspiration and severe respiratory challenges [176]. In cases of severe dysphagia and frequent aspiration, surgical interventions like gastrostomy placement may be considered [44]. For patients experiencing constipation and diarrhea, dietary modification can be considered as a first-line therapy [177]. However, if symptoms persist, the use of pharmacological therapy, including proton pump inhibitors, anti-dyspeptic drugs, metoclopramide and gentle laxatives, may be appropriate [112,178]. A recent study recommends early screening for GI and urological symptoms as part of standard care for children with DM1, stating that pharmacological treatment has shown excellent results in managing GI symptoms [179].

### 4.5. Central Nervous System

In DM1, CNS involvement is an important feature that impacts patients’ quality of life and prognosis. Cognitive deficits, behavioral changes and affective disturbances are predominant [180], with neuropathological findings in DM1 patients including neuronal loss and neurofibrillary aggregates, particularly affecting regions responsible for memory, motor control and emotional regulation [181,182,183]. The importance of CNS involvement underlines the need for a complete rehabilitation strategy to improve the quality of life of patients with DM1, beyond just medical care. Clinicians should frequently assess cognitive, behavioral and psychiatric symptoms, conducting detailed neuropsychological evaluation post-diagnosis [118]. Regular assessment of fatigue and sleep disturbances should be conducted using scales such as the daytime sleepiness scale (DSS), fatigue severity scale (FSS), DM1-specific fatigue and daytime sleepiness scale (FDSS), Epworth sleepiness scale (ESS) and excessive daytime sleepiness (EDS) evaluation, to monitor and manage these common symptoms [44,184]. Cognitive behavioral therapy (CBT) has been effective in reducing severe fatigue in DM1 individuals, resulting in improvements on daily activities and social involvement [185].

### 4.6. Visual System

Several ocular structures may be affected in DM1 patients, such as lens, retina, cornea, ciliary body, eye muscles and eyelids [186]. Common ocular symptoms include cataracts, ptosis, thicker corneas, macular alterations and decreased intraocular pressure (IOP), reticular maculopathies and Fuchs’ endothelial corneal dystrophy (FECD) [187,188]. Cataracts often present a distinctive Christmas tree shape and tend to progress early in patients with DM1, including in those with moderate disease signs [186]. FECD, which recently identified as an ocular manifestation in DM1, occurs in about 46% of patients and seems to be triggered by *DMPK* trinucleotide expansion through RNA-mediated toxicity [186,189]. Other ophthalmic manifestations include weakness in the orbicular muscles, epiretinal membranes, optic atrophy, poor adaptation to dim light, irregularities in iris pigment and alterations in the retinal pigment epithelium (RPE) [186].

Due to these risks, it is recommended that DM1 patients have yearly ophthalmologist visits, including a slit lamp examination [44,112]. Cataracts should be treated with surgical extraction when vision is impaired, and symptomatic oculomotor abnormalities, such as brow ptosis, can be corrected surgically if they interfere with daily activities. Also, eyelid crutches may be considered for patients undergoing ptosis [190]. Finally, to avoid misdiagnosing surgically treatable conditions such as epiretinal membrane, optical coherence tomography (OCT) is recommended for DM1 patients with visual impairments [191].

### 4.7. Endocrine System and Metabolism

Endocrine and metabolic abnormalities are well-known in DM1, with hyperinsulinemia following glucose ingestion often indicating prediabetes or impaired glucose tolerance [192]. This leads to a higher occurrence of thyroid, parathyroid and gonadal dysfunctions, as well as irregular adrenal hormone levels [97,193]. Initial evaluations should include hormonal status, covering sex hormones, thyroid and parathyroid hormones, lipid profiles and glucose metabolism [112,194]. It is also suggested to annually monitor the hemoglobin A1C (HbA1c) and fasting serum glucose levels [195].

To manage glucose metabolism disorder, therapy should follow established guidelines and the diagnostic criteria of the American Diabetes Association (ADA) [196]. Metformin is often considered the first-choice drug for DM1 patients with compromised glucose metabolism, as it effectively reduces blood glucose levels by suppressing hepatic gluconeogenesis and promoting glucose uptake [197,198]. Poorly treated diabetes mellitus can complicate the clinical scenario by causing diabetic polyneuropathy, which exacerbates gait instability and distal weakness [199]. Fatigue and muscle impairment can also be caused by hyperparathyroidism [200,201]. Implementing healthy lifestyle changes concerning diet and exercise, along with the appropriate use of various medications, can help regulate blood glucose and insulin levels, thereby addressing insulin resistance [202].

Recently, studies indicated that metabolome evaluation in these patients is crucial and may contribute to a better characterization and discrimination between DM1 disease phenotypes and severities [203]. Several experimental approaches using Fourier transform infrared spectroscopy (FTIR) allow the evaluation of metabolic profiles by categorizing samples through their biochemical composition. FTIR spectra were acquired and analyzed using skin DM1 patient-derived fibroblasts and controls. The results obtained showed clear discrimination between both DM1-derived fibroblasts with different CTG repeat lengths and with the age of disease onset. These results suggest that FTIR spectroscopy is a valuable tool for discriminating both DM1-derived fibroblasts with different CTG length and the age of onset and to study the metabolomic profile of patients with DM1 [203].

### 4.8. Reproductive System

DM1 patients often suffer fertility-related dysfunctions, with approximately 80% of affected males developing testicular atrophy by adulthood [204]. While less is known about female fertility in DM1, studies have shown a decreased in ovarian sensitivity [205,206,207], poor-quality embryos, and lower pregnancy rates among female patients [208,209]. In rare cases, premature ovarian failure may occur [210]. Maternal complications during pregnancy can include prolonged labor due to uterine dysfunction, uterine overdistention linked with polyhydramnios and post-partum hemorrhage developing from atonic uterus [211,212,213]. Fertility issues in DM1 should be assessed through blood tests to assess reproductive hormones and semen evaluation for sperm count and quality [204,214].

While no effective treatments are available to restore fertility, assisted reproductive technology (ART), with or without oocyte or sperm donation, may be beneficial to improve the chance of conception [215]. Intensive obstetric and perinatal care is strongly recommended due to the increased risks, particularly during pregnancy and delivery [216,217]. In cases of polyhydramnios, amniotic fluid volume reduction can be considered to manage preterm labor or substantial maternal distress, using amnioreduction and/or prostaglandin synthetase inhibitors [218,219]. Future research is essential to improve treatments and outcomes for reproductive issues in patients with DM1.

### 4.9. Integumentary System

DM1 patients often present skin-related abnormalities, such as frequent dysplastic nevi, xerosis, alopecia and seborrheic dermatitis, beside premature aging signs [220]. These patients commonly exhibit skin abnormalities, with the most common cases primarily related to the severity of the genotype and serum vitamin D levels. An increase in nevi count is associated with larger CTG expansions, while both dysplastic nevi and xerosis are linked to lower vitamin D levels [221]. While limited research exists on skin involvement in DM1, associations have been observed with baldness (early male frontal alopecia) [222], epithelial tumors (pilomatricomas and non-melanoma skin cancers (NMSC)) [223]. Pilomatricomas are firm lumps that often appear near the head or neck below the skin’s surface [224]. Isolated pilomatricomas may appear during childhood and could be an early sign of the disease, which should be first recognized by pediatric dermatologists [44], as it can be effectively treated by surgical removal. While skin changes have been reported with DM1, the specific mechanism of the disease in causing these alterations is still uncertain. Identifying dermatological markers of DM1 could further provide clinicians with valuable insights.

## 5. Towards the Development of Promising Therapeutic Interventions for DM1

In recent years, emerging studies in DM1 focused on addressing the involved molecular mechanisms and on exploring the generation of therapeutic approaches, which collectively could develop more effective and long-lasting treatments [225,226].

Disruptions caused by the expansion of CTG trinucleotide repeats in the *DMPK* gene underlie the widespread splicing defects and cellular dysfunctions related to DM1. Consequently, developing therapies for DM1 requires a deep understanding of its molecular pathology, which has led the identification of key therapeutic targets [227], paving the way for drug development and advancing promising candidates through clinical trials to assess their safety and effectiveness.

**Figure 4:**
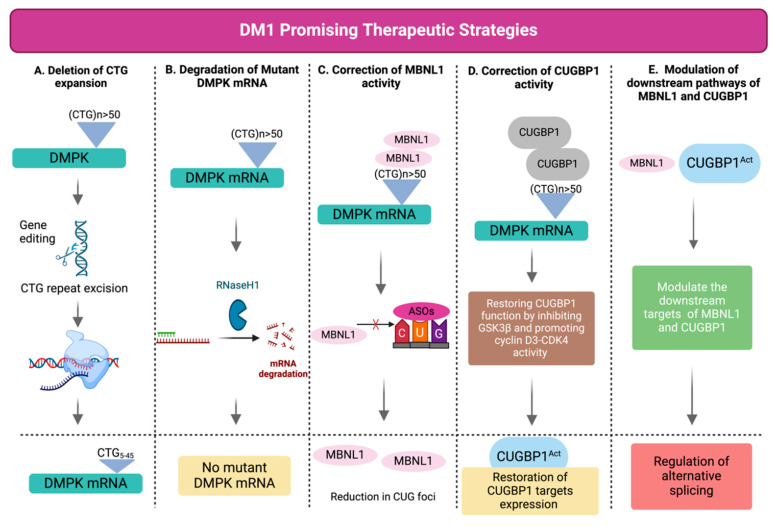
The main therapeutic interventions proposed for DM1. This figure illustrates the five potential therapeutic approaches already described, which target the molecular mechanisms underlying DM1. (**A**) CTG repeat excision, targets the root cause of DM1 by using gene-editing technologies to eliminate the repeat expansions in the DMPK gene. (**B**) RNaseH1-mediated degradation of mutant DMPK mRNA transcripts to prevent toxic RNA foci formation. (**C**) Mutant RNA degradation, uses antisense oligonucleotides (ASOs) or small interfering RNAs (siRNAs) to degrade toxic RNA species, alleviating downstream effects associated with the disease. (**D**) Restoration of normal CUGBP1 activity by targeting upstream regulators like GSK3β (**E**) Modulation of the downstream targets of MBNL1 and CUGBP1 to correct disease-related splicing changes [229,241,242]. Figure created with BioRender (BioRender.com), and adapted with permission from [243], licensed under CC BY 4.0 (https://creativecommons.org/licenses/by/4.0/) Abbreviations: DMPK, myotonic dystrophy protein kinase; (CTG)n, expansion of (cytosine-thymine-guanine) n; mRNA, messenger ribonucleic acid; CELF, CUGBP Elav-like family; MBNL1, muscleblind-like splicing regulator 1; GSK-3β, glycogen synthase kinase-3 beta.

A major therapeutic approach strategy already proposed for DM1 involves using CRISPR-Cas9 technology to excise the expanded CTG repeats from the *DMPK* gene. This strategy focuses on the removal of the expanded CTG repeats from the mutant *DMPK* gene, efficiently correcting the mutation at the DNA level. By targeting the root cause of DM1, CRISPR-Cas9 can effectively target and excise the repeat expansions in cell models, restoring normal DMPK function [228] (Figure 4A).

In addition to gene editing, RNA-targeting therapies have emerged as an important area of focus. Degrading mutant DMPK mRNA in patient tissues using antisense oligonucleotides (ASOs) and small interfering RNAs (si-RNAs) have been developed, triggering transcript degradation mediated by RNase H for ASOs or the RNA-induced silencing complex (RISC) for siRNAs. By eliminating the mutant mRNA, these therapies aim to restore the normal function of MBNL1 and reduce toxic foci formation, leading to improved cellular function and splicing outcomes [229,230] (Figure 4B). Recent studies have shown the potential for synergistic effects when combining ASOs with small molecules that target splicing regulators. These combinatorial strategies enhance therapeutic efficacy by presenting several molecular defects in DM1 [62,231]. MBNL1, which is typically sequestered by toxic RNA, can be used to reduce CUG foci accumulation. This sequestration disrupts normal splicing and gene expression processes, leading to misregulated splicing of critical genes involved in muscle function and other cellular processes [65,232]. Specifically, MBNL1 is responsible for regulating alternative splicing events that are crucial for muscle fiber composition and function. When sequestered by toxic RNA foci, MBNL1 cannot perform its normal regulatory functions, resulting in aberrant splicing patterns that contribute to muscle degeneration and dysfunction [233] (Figure 4C).

Another promising approach involves targeting the downstream effects of RNA toxicity at the protein level. The dysregulation of CUGBP1, a critical RNA-binding protein, contributes to the disruption of splicing and gene expression in DM1 [234]. Therapeutic strategies aimed at modulating CUGBP1 activity have shown promise in restoring normal RNA splicing patterns. For instance, the inhibition of GSK3β, cyclin D3, and CDK4 to restore the function of CUGBP1, helps to maintain the effective function of RNA splicing and degradation of mutant DMPK mRNA [70,235] (Figure 4D).

Another strategy involves therapeutics that correct downstream targets of both MBNL1 and CUGBP1 and could be used for DM1 therapy (Figure 4E). Insulin receptor (IR) is among the first identified splicing targets of CUGBP1 and MBNL1 that is misregulated in DM1 [236,237]. IR seems to be involved in DM1 pathogenesis as this disorder exhibits insulin resistance and predisposition to type 2 diabetes (T2D) [238,239]. It was shown that the diabetes drug, metformin, corrects abnormal IR splicing in DM1 mesodermal precursor cells (MPCs) and in DM1 myoblasts [197,240], suggesting possible therapeutic benefits (Figure 4E).

As research continues to advance the understanding of DM1’s complex molecular mechanisms, innovative therapeutic approaches provide a multifaceted strategy that holds promise for more effective treatments. These advancements, summarized in Table 1, highlight important investigational therapies designed to address pathological features of DM1. Furthermore, many of these compounds are currently undergoing clinical trials, each contributing uniquely to the growing arsenal of therapeutic options.

### 5.1. Small Molecules

The exploration of small molecules as potential therapies for DM1 is being investigated to address the genetic and molecular defects in DM1. The theoretical advantages of these small molecule compounds include broad biodistribution as well pharmacokinetics and pharmacodynamics in humans, lower manufacturing costs and the ease of oral administration, a feature required for treating the multisystem nature of the disease [244]. Hence, nearly all identified molecules are repurposed compounds targeting various disease mechanisms, which represent the leading strategies for clinical approval (Table 1).

One of the most promising small molecules is Tideglusib (AMO-02), a GSK3β inhibitor [245]. Increased activity of the protein kinase GSK3β has been found in experimental animal models and muscle samples from DM1 patients, particularly in the congenital and adult forms of DM1 [235,246]. By targeting GSK3β, Tideglusib impacts skeletal muscle and brain defects through several pathways [225,247,248]. Developed by AMO-Pharma, Tideglusib completed a Phase II clinical trial (NCT02858908) in both congenital and childhood-onset DM1, with most participants experiencing improved CNS and clinical neuromuscular symptoms [249]. Due to its favorable pharmacokinetic and safety profile and significantly reduced myotonia in half of the subjects, a subsequent open-label Phase II/III was announced in 2021 to investigate its long-term safety and efficacy (NCT03692312, and NCT05004129, Table 1).

Erythromycin, a commonly prescribed antibiotic for chronic obstructive pulmonary disease (COPD), has also shown therapeutic potential in DM1. Preclinical research showed that erythromycin can suppress RNA toxicity and correct splicing abnormalities in DM1 cells and mouse models [250,251]. At COPD-equivalent doses, erythromycin displayed beneficial effects on DM1 mice, prompting further investigation in clinical trials [228,252,253,254]. Building on these findings, a Phase II study clinical trial (JPRN-jRCT2051190069) sponsored by Osaka University Hospital, was initiated to evaluate the safety and effectiveness of erythromycin (MYD-0124) in DM1 patients (Table 1). The trial found that erythromycin was safe and well-tolerated, showing potential as a therapeutic option for DM1 [251].

Metformin, a biguanide antidiabetic drug, has also been proposed as a promising therapy for DM1 due to its ability to correct metabolic and mitochondrial defects in patient-derived fibroblasts [68]. The results of a Phase II clinical trial (2013-001732-21) indicated that while oral metformin improved DM1 patients’ mobility, it did not affect myotonia or muscle weakness compared to the control group (Table 1). Currently, an ongoing Phase III replication study (2018-000692-32) sponsored by Vergata University of Rome is evaluating metformin’s effects in adult DM1 patients with expectations to deliver approvals on this agent on its use as a potential therapy (Table 1). The announcement of a new Phase III trial (NCT05532813) by Assistance Publique—Hôpitaux de Paris aims to assess the motor improvements in non-diabetic DM1 patients (Table 1).

Mexiletine is safe for short-term use and effective in reducing handgrip myotonia in DM1 [111,255]. The completed Phase II trial (NCT01406873) administered mexiletine (brand name NaMuscla^®^) orally to assess its effectiveness in managing myotonia symptoms in adult DM1 patients (Table 1). While the trial indicated improvements in handgrip myotonia, it did not demonstrate significant benefits in the 6 min walk test (6 MWT). Sponsored by Lupin, two Phase III trials were launched [228,253,254] in 2021 to further assess mexiletine’s impact, including a study involving children and adolescents (NCT04624750). The second trial (MIND Study; NCT04700046), involving DM1 adults, was withdrawn. Other ongoing study trials (NCT04622553, NCT04616807) are evaluating the long-term effectiveness of mexiletine, with results expected in 2026 (Table 1).

Furthermore, ERX-963 (Flumazenil), a repurposed GABA_A receptor antagonist originally used to reverse benzodiazepine overdose, was evaluated in a crossover study (NCT03959189). While the compound demonstrated safety, it failed to improve measures of sleepiness or vigilance in the trial [256]. Meanwhile, other compounds, such as Ranolazine and Pitolisant are being explored to address DM1 symptoms like myotonia, chronic pain and daytime sleepiness (Table 1). A clinical trial (NCT04634682) evaluated the impact of a nutritional approach combining theobromine and caffeine (marketed as MYODM^TM^ by Myogem Health Company) for its potential to improve quality of life, reduce fatigue and alleviate hypersomnia in adults with DM1 (Table 1).

Finally, a preclinical study used senolytics, in particular the BCL-XL inhibitor (A1155463; small molecule), and demonstrated that it can specifically remove senescent DM1 myoblasts by inducing their apoptosis and rescuing their proliferation and differentiation capacity. These findings identify the pathogenic mechanism associated with muscle stem cell defects in DM1 and open a therapeutic possibility that targets these defective cells to restore myogenesis [257].

**Table 1:** Novel therapeutic approaches for DM1 targeting specific disease symptoms. Overview of specific molecules, their targets, clinical development status, modes of action, the physiological systems and symptomatic domains (e.g., neuromuscular, respiratory, cognitive) targeted or improved by these therapies. This table categorizes therapeutic agents based on their intervention in disease mechanisms to alleviate DM1 symptoms.

Class	Drug Candidate (Compound)	Clinical Trials Registry Identifier; Clinical Trial Phase	Mode of Action (MoA)	Study Design	Body Systems/Outcomes Improved	References
**Repurposed small molecule**	Tideglusib (AMO-02)	NCT02858908; Phase II	Inhibition of GSK3β activity	- Randomized, single-blind study with 16 participants aged 13–34 with congenital and childhood-onset DM1 - Participants received a fixed oral dose of either 400 mg or 1000 mg of AMO-02.	- Improvements in CNS function and clinical neuromuscular symptoms in most patients - Favorable PK profile	[249,258]
		NCT03692312; Phase II/III	Inhibition of GSK3β activity	- Randomized, placebo-controlled study (REACH CDM) in children and adolescents (56 participants) with congenital DM1 (aged 6–16 years) - Participants received 1000 mg/day of Tideglusib compared to a placebo.	- Tideglusib demonstrated promising secondary outcomes - Improvements in motor function (10 m walk/run), cognitive performance (Peabody Picture Vocabulary Test), and muscle integrity, indicated by reduced creatine phosphokinase (CPK) levels - Tideglusib was well tolerated, with a favorable safety profile and no treatment-related serious adverse events.	[259]
		NCT05004129; Phase II/III	Inhibition of GSK3β activity	- Open-label, 52-week extension study (REACH CDM X) in children and adolescents with congenital or childhood-onset DM1 - The planned enrolment was increased from 56 to 76 participants - Participants received 1000 mg/day of Tideglusib - Included both treatment-naïve individuals and those from the AMO-02-MD-2-003 study for continuity, long-term follow up - An option for extended access was offered	- Improved motor function, myotonia, and respiratory function	[260,261]
	Erythromycin (MYD-0124)	JPRN-jRCT2051190069; Phase II	Reduction of RNA toxicity	- Randomized, double-blind, placebo-controlled study (MYD-0124) - A total of 30 adult patients with DM1 were enrolled and randomly assigned - Patients received 500 mg/day or 800 mg/day	- Demonstrated favorable safety and tolerability profiles	[251]
	Metformin	2013-001732-21; Phase II	AMPK activation	- Randomized, double-blind, placebo-controlled study with 40 DM1 patients (aged 18 and 60 years) - To evaluate the efficacy of metformin on ambulation in DM1 patients	- Statistically significant improvement in mobility and total mechanical power	[262]
		2018-000692-32; Phase III	AMPK Activation	- Randomized, double-blind, placebo-controlled study - For the 23/40 adult DM1 patients who fully completed the 1-year study to assess the impact of metformin on motility and strength	- Improved the 6 min walk test (6 MWT)	[262]
		NCT05532813; Phase III	AMPK Activation	- Multicenter, randomized, double-blind, placebo-controlled trial (METFORMYO) to evaluate the efficacy and safety of metformin in treating DM1	- Specifics on results not provided	[263]
	Mexiletine	NCT01406873; Phase II	Na^+^ channel blocker	- Randomized, double-blind, placebo-controlled study to assess the efficacy of mexiletine on ambulatory function and its long-term safety in (aged 18–80 years) DM1 adults - Received 150 mg/kg mexiletine in capsules taken orally, three times daily for 6 months or 150 mg/kg placebo capsules taken orally, three times daily for 6 months	- Improved handgrip myotonia force relaxation time at 6 months - No significant effect on the 6 MWT was recorded	[110]
		NCT04624750; Phase III	Na^+^ channel blocker	- Open-label, multicenter, single-arm study in pediatric DM1 patients (aged 6–18 years) - Evaluated the pharmacokinetics, safety, and efficacy of mexiletine	- Evidence of long-term drug efficacy and cardiac safety	[264,265]
		NCT04622553; Not Applicable	Na^+^ channel blocker	- Open-label extension study assessing the long-term safety and efficacy of mexiletine in pediatric patients with myotonic disorders	- Mexiletine was well tolerated in the study - Specifics on results not provided	[266]
		NCT04616807; Not Applicable	Na^+^ channel blocker	- Observational study evaluating the long-term safety and effectiveness of mexiletine (NaMuscla) in managing myotonia in adult patients with non-dystrophic myotonic disorders	- Improved myotonia	[267]
		NCT04700046; Phase III (Withdrawn)	Na^+^ channel blocker	- Randomized, double-blind, placebo-controlled, multicenter trial (MIND) assessing the efficacy and safety of mexiletine over 26 weeks in DM1/DM2 patients	- Demonstrated efficacy of mexiletine in patients with adult-onset DM1 - Improved hand-grip force relaxation time at 6 months, - No significant effect on the 6 MWT was recorded	[110,268]
	Flumazenil (ERX-963)	NCT03959189; Phase I	GABA receptor antagonist	- Double-blind, placebo-controlled, dose-ranging crossover study assessing the safety and tolerability of a single-day administration of ERX-963 to reduce daytime sleepiness and enhance cognition (18 to 65 years) in DM1.	- ERX-963 at 1 mg and 2 mg doses was safe and well tolerated - No evidence of efficacy in measures of sleepiness or vigilance at the tested doses and administration regimen	[269]
	Ranolazine	NCT02251457; Phase I	Inhibition of late sodium current	- Open-label study evaluating the tolerability and effects of ranolazine in DM1 patients - Participants assessed at baseline, after 2 weeks of 500 mg ranolazine twice daily, and after 4 weeks on 1000 mg twice daily - Prior mexiletine users were evaluated before discontinuation	- Significant improvement in clinical myotonia	[270,271]
	Pitolisant	NCT04886518; Phase II	H3 receptor antagonist	- Randomized, double-blind, placebo-controlled study to evaluate the efficacy of Pitolisant in treating excessive daytime sleepiness and other non-muscular symptoms in (aged 18 to 65 years) DM1 patients	- Improved excessive daytime sleepiness (EDS) and fatigue	[272]
**Repurposed small molecule** **(Natural compounds)**	Theobromine and caffeine (MYODM™)	NCT04634682; Not Applicable	Reduction of toxic DMPK RNA	- Evaluation of MYODM™ (caffeine/theobromine supplement) as a food supplement in 30 adults with DM1.	- Improved quality of life in adult males with DM1 - Statistically significant decrease in the Epworth sleepiness scale and increase in 6 MWT results	[273]
**Antibody fragment conjugated antisense oligonucleotide (ASO)**	DYNE-101 (TfR1-targeted antibody (Fab) linked to ASO)	NCT05481879; Phase II/III	Reduction of toxic DMPK RNA	- Randomized, placebo-controlled trial (ACHIEVE) evaluating the safety, tolerability, pharmacodynamics (PD), efficacy and pharmacokinetics (PK) of multiple ascending doses of DYNE-101 in adults with DM1	- Improved myotonia and muscle strength - Favorable safety profiles	[274,275]
	IONIS-DMPKRx (Baliforsen; ISIS 598769)	NCT02312011; Phase I/IIa	Reduction of toxic DMPK RNA	- Blinded, placebo-controlled, dose-finding trial evaluating the safety, tolerability, and dose range of multiple ascending subcutaneous doses of Baliforsen in adult DM1 patients.	- Baliforsen was generally well tolerated - Skeletal muscle drug concentrations were below levels anticipated to achieve substantial target reduction	[276]
**Anti- microRNA oligonucleotide**	ARTHEx’s ATX-01	NCT06300307; Phase I/IIa	Anti-microRNA, MBNL upregulation	- Double-blind, placebo-controlled trial in adults with classic DM1 (aged 18–64) evaluating the safety, tolerability, PK, PD and efficacy of single and multiple intravenous doses of ATX-01	- Effectively targets many affected tissues, enhancing therapeutic efficacy and safety	[277]
**Antibody oligonucleotide** **conjugated**	MARINA AOC 1001	NCT05027269; Phase I/II	TfR1-targeted antibody (mAb) conjugated to siRNA targeting DMPK	- Randomized, double-blind, placebo-controlled clinical trial (MARINA^®^) that enrolled 38 adults with DM1 - Evaluating the safety and tolerability of single and multiple ascending doses of del-desiran administered intravenously	- Improved myotonia - DMPK reduction observed	[278,279]
	AOC 1001	NCT05479981; Phase II	TfR1-targeted antibody (mAb) conjugated to siRNA targeting DMPK	- Open-label, multicenter trial (MARINA-OLE™) designed to evaluate the long-term safety and tolerability of del-desiran in participants with DM1 who were previously enrolled in the MARINA^®^ Phase 1/2 trial - This trial will monitor the long-term safety, tolerability, efficacy, PK, and PD of del-desiran in adults with DM1	- Ongoing assessment of myotonia, hand function, strength and mobility	[280]
	Del-desiran (Delpacibart Etedesiran), previously AOC 1001	NCT06411288; Phase III	TfR1-targeted antibody (mAb) conjugated to siRNA targeting DMPK	- Randomized, placebo-controlled, double-blind pivotal study (HARBOR™) designed to evaluate del-desiran in approximately 150 people (aged 16 and older) living with DM1 - Patients will be administered either del-desiran or placebo (1:1) every eight weeks - The trial is designed to assess del-desiran’s impact on multiple key aspects of DM1 including myotonia, muscle strength and activities of daily living.	- Specifics on results not provided	[281,282]
**Peptide conjugated antisense oligonucleotide (ASO)**	PGN-EDODM1	NCT06204809; Phase I	RNase H-mediated degradation of target RNA	- Randomized, placebo-controlled (FREEDOM-DM1) trial exploring the safety, tolerability, PK and PD of single ascending doses of PGN-EDODM1 in adults with DM1	- Nonclinical pharmacology studies with PGN-EDODM1 showed considerable therapeutic potential for splicing correction and myotonia improvement	[283,284]
**Small interfering RNAs (siRNA) conjugates**	ARO-DM1	NCT06138743; Phase I/IIa	RNA interference (RNAi) technology targets specific mRNA molecules	- Randomized, double-blind, placebo-controlled study in adults with DM1 (aged 18–65) evaluating the safety, tolerability, PK and PD of ARO-DM1	- Preclinical data show ARO-DM1 reduces muscular DMPK expression and corrects spliceopathy, potentially improving muscle strength, function, and mobility	[285]
**Peptide conjugated oligonucleotide**	VX-670	NCT06185764; Phase I/II	Degrades the toxic DMPK	- Randomized, double-blind, placebo-controlled design evaluating the safety, tolerability, PK, and PD of single and multiple intravenous doses of VX-670 in adults with DM1	- Specifics on results not provided	[286]

CNS, central nervous system; PD, pharmacodynamics; PK, pharmacokinetics.

### 5.2. Nucleic Acid-Based Therapies

Nucleic acid-based therapies, recognized as the third major drug class alongside small molecules and antibodies, use DNA or RNA as active components to treat various diseases, including DM1 [287]. These innovative therapies encompass a range of technologies such as antisense oligonucleotides (ASOs), small interfering RNA (siRNA) and CRISPR-Cas9, providing powerful tools for disease intervention. Among these, oligonucleotides—small synthetic nucleic acid polymers (~20-m), either single- or double-stranded—modulate gene expression through diverse mechanisms, including inducing RNA degradation, altering splicing events, or inhibiting protein translation by targeting pre-mRNA, mRNA, or non-coding RNA [288].

### 5.3. Antisense Oligonucleotides (ASOs)

ASOs, are known to bind specifically to mRNA transcripts, leading to their degradation or the modification of their function [289]. Upon binding, they can induce mRNA degradation or alter splicing patterns to produce functional proteins. The ACHIEVE study, sponsored by Dyne Therapeutics, proposes a strategy that enhances targeted tissue drug delivery by using a fragment of monoclonal antibodies conjugated (mAb) to a transferrin receptor 1 (TfR1) with ASOs; the study is assessing DYNE-101 (FORCE-DMPK) in an ongoing Phase II/III clinical trial (NCT05481879). DYNE-101 has demonstrated 40–50% splice-correction efficacy in preclinical studies by significantly reducing toxic DMPK RNA in skeletal and cardiac muscle tissues with no adverse effects after 13 weeks in cynomolgus monkeys [290]. The objective of the trial is to investigate the safety, tolerability, pharmacodynamics (PD), efficacy and pharmacokinetics (PK) of escalating doses of DYNE-101 in DM1 patients (Table 1). Similarly, IONIS Pharmaceuticals designed IONIS-DMPKRx (baliforsen; ISIS 598769), which underwent a Phase I/IIa study (NCT02312011). While baliforsen was found to be safe and tolerable, it did not achieve therapeutic ASO levels in skeletal muscle at the maximum tested dose (600 µg).

ARTHEx’s ATX-01 is an oleic acid-conjugated antimiR oligonucleotide with preferential delivery to target tissues (muscle and brain) designed to inhibit microRNA 23b (miR-23b), which is a natural repressor of MBNL protein expression [291,292,293]. The first human trials (NCT06300307) of ATX-01 in participants with classic DM1 are ongoing, with initial safety and efficacy data anticipated soon (Table 1). PepGen Inc is currently investigating PGN-EDODM1 through a randomized, placebo-controlled, phase 1 FREEDOM-DM1 trial (NCT06204809). This ASO is conjugated to a cell-penetrating peptide for enhanced delivery, targeting and neutralizing toxic CUG repeats in DMPK transcripts [242,294,295]. Likewise, a Phase II study (FREEDOM2) is planned to evaluate the safety and therapeutic effects of multiple ascending doses in adult DM1 patients, subject to regulatory clearance [296].

### 5.4. SiRNA-Based Therapies

Avidity Biosciences launched the MARINA™ Phase I/II trial (NCT05027269), which was completed in 2023. The primary objective of this trial was to assess the safety, tolerability, PK and PD of the intravenously injected AOC 1001, which combines siRNA and fragment antigen-binding region (Fab region) to target transferrin-receptor 1 (*TfR1*). A subsequent Phase III extension study (NCT05479981) is evaluating its long-term efficacy in improving hand myotonia, muscle strength and daily function. The HARBOR™ Phase 3 trial (NCT06411288) is an international, randomized, placebo-controlled study focused on del-desiran’s safety and efficacy in approximately 150 DM1 individuals (aged 16 and older) with DM1 (Table 1).

### 5.5. RNAI and Phosphorodiamidate Morpholino Oligomers (PMO) Therapeutics

Arrowhead Pharmaceuticals’ ARO-DM1, an RNAi therapeutic targeting the DMPK gene, has shown preclinical efficacy, with an 80% reduction in DMPK mRNA in non-human primate muscles (NCT06138743; Phase I/IIa). Preclinical evidence demonstrated an 80% reduction in DMPK mRNA levels in non-human primate skeletal muscles, with effects lasting over 85 days. This approach aims to improve muscle strength and function by addressing the expanded CUG repeats in the DMPK transcript’s 3’-UTR. In DM1 mice model, the species-specific S-ARO-DM1 variant reduced DMPK-CUG expression and fixed abnormal splicing [285]. VX-670 (NCT06185764; Phase I/II), a phosphorodiamidate morpholino oligonucleotide (PMO) developed by Entrada Therapeutics and licensed to Vertex, combines a cyclic peptide for efficient delivery to muscle nuclei. VX-670 interacts with the CUG repeat RNA to release MBNL1, aiming to correct mis-splicing defects central to DM1 pathology. Its innovative design may overcome the several delivery challenges faced by oligonucleotides in muscle tissue [297]. To be effective, these treatments often require high and frequent dosing, and must be injected repeatedly unless injected directly into the target tissue, due to the restricted biodistribution [286,298] (Table 1).

### 5.6. Genome/Transcriptome Engineering Approaches

Gene therapy strategies, particularly those utilizing adeno-associated virus (AAV) vectors, aim to deliver functional copies of genes or modify gene expression directly within target tissues.

To overcome limitations and challenges associated with ASOs, AAV-based gene therapy strategies are being evaluated for DM1 [299], as the success of Zolgensma gene replacement approach in spinal muscular atrophy (SMA) has paved the way for related approaches [300]. Astellas Gene Therapies is developing AT466, an AAV vector designed to deliver a functional *MBNL1* gene to restore normal protein expression and to prevent toxic RNA aggregate accumulation [301]. CRISPR-Cas9 technology offers promising possibilities for DM1 treatment by removing the pathogenic CTG expansion from the *DMPK* mutant allele or using a nuclease-free derivative to slow *DMPK* transcription by targeting the CTG repeats. RNA-targeting Cas9 could directly degrade the toxic CUGexp-DMPK transcript by targeting the CUG repeats, exhibiting an assuring avenue for managing the disease [302]. Two promising approaches that involve AAV vectors expressing CRISPR-Cas9 (SaCas9) and AAV-vectors encoding PIN-dCas9 (a nuclease dead Cas9 (dCas9)) are proceeding to preclinical stages [303]. In DM1, AAV-SaCas9 can be utilized to precisely target and excise the CTG repeat expansions in the *DMPK* gene [304]. Meanwhile, AAV-vectors encoding PIN-dCas9, developed by LocanaBio, specifically bind and degrade toxic CUG-repeat RNA molecules implicated in diseases such as DM1. By avoiding DNA cleavage, this minimizes off-target effects and preserves genomic integrity [305]. AAV9 vectors with the selected leads (A01215, A01344, A01686) are being tested in non-human primates, using muscle-specific promoters to evaluate tolerability and transgene expression in skeletal, smooth and cardiac muscles [306].

Another innovative gene therapy involves bio-engineering RNA-binding decoy proteins with high affinity for pathogenic repeats in mutated RNA, liberating MBNL1 proteins to resume their normal regulatory roles [307]. While further preclinical development is necessary, including studies on dosage, toxicity, sponsor selection and AAV vectors, challenges persist. These include immunogenicity (innate, cellular and humoral), off-target gene editing, limited cargo capacity, tissue-specific transduction delivery techniques and ethical concerns, all of which must be tackled before clinical trials can advance [308,309]. Researchers at the Institute of Myology, in collaboration with teams from the University of Liège, developed a gene therapy using CRISPR interference (CRISPRi) to silence the *Dmpk* gene in DM1 mice, achieving over 80% of gene silencing and the correction of disease-related abnormalities [310,311]. This method’s specificity suggests that CRISPRi could be a promising therapeutic approach for DM1, minimizing off-target side effects [312].

## 6. Challenges and Future Directions

The complex and heterogeneous nature of DM1 presents several challenges for therapeutic interventions, primarily due to its impact on several body systems [36]. The disorder’s phenotypic variability, ranging from myotonia and mild muscle weakness to severe multisystem complications involving the endocrine, cardiac and neurological systems, make the development and standardization of effective treatments difficult [12,313]. This disease heterogeneity requires therapies that address not only the primary molecular pathology but also the diverse downstream effects of the disease.

Several potential treatments, such as small molecules, ASOs and CRISPR/Cas9 technology, are emerging with promising results. However, these approaches face hurdles with delivery efficiency and tissue specificity, with overcoming the blood–brain barrier and also the diversity of tissues affected by DM1 [314,315]. Nanoparticle-based approaches and advanced viral vectors are under active investigation to enhance delivery to specific tissues while minimizing off-target effects [316,317,318].

Although research into cell therapy is still in its nascent stages, promising avenues are being explored [228,254]. For instance, investigations into donor-derived engrafted cells reveal that they may develop toxic RNA foci through MBNL1 sequestration and abnormal splicing. This phenomenon suggests that harmful CUG repeat RNA could migrate from the original nucleus to other nuclei within the myofiber, further exacerbating pathology [226].

Conducting clinical trials for rare diseases like DM1 are far more complex than for common conditions due to the unmet need for effective treatments and limited research results [319]. A major hurdle specific to DM1 is the limited number of eligible trial participants and the geographical dispersion of patients. This dispersion reduces patient access to centralized trial sites and reduces the statistical power needed to identify differences between treatment and control groups. Phenotypic variability and genetic anticipation—where disease severity increases and onset age decreases in successive generations—necessitate longitudinal studies with extended follow-up periods to assess treatment efficacy and disease progression [43,320]. This variability in clinical appearances can result in inconsistent responses to treatments, complicating both the assessment of treatment efficacy and the discovery of effective biomarkers. A thorough understanding of the RNA toxicity, splicing dysregulation and protein sequestration is important for advancing research in DM1, but these areas remain under active investigation [321].

Standardizing outcome measures, including biomarkers and patient-reported outcomes, is critical to overcoming inconsistencies in clinical responses and ensuring robust evaluations of therapeutic efficacy [322,323]. Engaging a multidisciplinary team, including endocrinologists, neurologists, cardiologists and other specialists experienced in DM1 management, is vital for enhancing both patient outcomes and improving quality of life. Furthermore, fostering international cooperation and data sharing could expedite the development of effective therapies while providing valuable insights into the global impact and incidence of DM1.

## 7. Conclusions

In conclusion, while a definitive cure for DM1 remains elusive, the management of its complex and multisystemic symptoms through supportive care has provided significant patient well-being. Further research is required to better understand the symptom burden and its effect on patients’ lives, as well as to develop approaches to alleviate them. A deeper understanding of the disease’s symptom burden and its impression on patients’ daily lives is central for refining care strategies. Implementing precise standards of care for symptom management and the early detection of comorbidities is fundamental, as evidence suggests that these measures can significantly improve patients’ quality of life. The active clinical trials and research initiatives underscore a promising array of potential treatments, offering optimism for an effective therapy for DM1 in the near future.

## Data Availability

Data are contained within the article. Further inquiries can be directed to the corresponding author.
